# Evaluation of biofilm accumulation on and deactivation force of orthodontic Ni-Ti archwires before and after exposure to an oral medium: A prospective clinical study

**DOI:** 10.34172/joddd.2020.006

**Published:** 2020

**Authors:** Diogo M. Sapata, Adilson L. Ramos, Sérgio Sábio, David Normando, Renata C. Pascotto

**Affiliations:** ^1^Department of Dentistry, State University of Maringá, Maringá-Pr, Brazil; ^2^School of Dentistry, Federal University of Pará, Belém-PA, Brazil; ^3^Department of Dentistry, State University of Maringá, Maringá-Pr, Brazil

**Keywords:** Modulus of elasticity, nickel, orthodontics, orthodontic wires, surface properties

## Abstract

***Background.*** This in vitro study aimed to evaluate biofilm accumulation on and deactivation force of orthodontic nickeltitanium (NiTi) archwires before and after exposure to an oral medium.

***Methods.*** Four commercial brands of orthodontic NiTi 0.016" archwires were examined before and after exposure to the oral medium for 4 weeks. Six archwire segments, 30 mm in length, from each manufacturer were tested in a device with four selfligating brackets, channel 0.022", adapted to a universal test machine to evaluate the deactivation force between 0.5 and 3 mm of deflection. The presence of biofilm on the archwire surfaces was evaluated by scanning electron microscopy, before and after exposure to the oral medium. The Wilcoxon and kappa tests were applied to the biofilm scores, three-way ANOVA for repeated measures (Bonferroni post-test), and linear regression between biofilm and deactivation force.

***Results.*** The exposure to the oral medium promoted moderate to severe presence of debris on the archwire surfaces and caused a reduction in deactivation force for the Ormco and GAC brands, while maintaining them with adequate force levels. The MORELLI and ORTHOMETRIC archwires underwent no significant reduction in deactivation force; moreover, these maintained elevated levels of force after exposure to the oral medium. The Spearman test indicated a low correlation between biofilm accumulation and deflection force for the Morelli (R2=0.132 and P=0.683) and Orthometric (R2=0.308 and P=0.330) brands. On the other hand, the GAC (R=0.767 and P=0.004) and ORMCO (R=0.725 and P=0.008) brands exhibited statistically significant correlation between these variables.

***Conclusion.*** Exposure to the oral medium for one month might give rise to significant changes in the dissipation of forces of orthodontic NiTi archwires, resulting from biofilm accumulation.

## Introduction

The properties of NiTi wires are responsible for promoting low forces at physiological thresholds at the beginning of orthodontic treatment, improving in efficiency during the treatment.^1- 2^ Deactivation force and surface roughness are outstanding among these properties because they influence adequate sliding of the wire in the bracket channel, and its deflection throughout orthodontic movement.^3-7^

Laboratory studies simulate orthodontic movements and reveal the different behaviors of archwires. However, there has been little investigation into the influence of biofilm accumulation, which might compromise the deactivation force of archwires when in contact with brackets, as a result of the greater degree of friction, thus interfering with the effectiveness of movements.^8-19^

In this sense, there are few trials about aging in the oral medium,^14,15^ and they point out interference with friction and deflection of archwires. However, up to now, little is known about the relationship between biofilm accumulation and the performance of NiTi archwires.

Eliades et al^19^ demonstrated that orthodontic materials in the oral cavity might perform differently from their as-received or in-vitro-agedcounterparts, and their properties might not correspond with what is specified by the manufacturer. Clinicians should understand the limitations and the reactions of materials after their exposure to the oral cavity medium.

This study evaluated the mechanical behavior of NiTi archwires before and after their exposure to the oral medium.

## Methods

The present study was approved by the Research Ethics Committee of the State University of Maringá (UEM) (CAAE: 43121915.2.0000.0104), and the subjects signed a term of free and informed consent.

This in vitro study aims to evaluate the mechanical behavior of NiTi archwires before and after their exposure to the oral medium.

Pre-shaped orthodontic NiTi archwires (0.40 mm, 0.016”) of four commercial brands were used as follows: Superelastic NiTi (Morelli, Sorocaba, Brazil), Flexy NiTi - Super Elastic (Orthometric, Marília, Brazil), Nitinol Archwire (GAC International, Bohemia, USA), Damon Optimal Force Copper NiTi (Ormco Corp., Glendora, USA), with six archwires each, three maxillary and three mandibular, with different lots between the arches, for the in vitro tests before and after aging in the oral cavity. The sample size was calculated at α=95% and β=80%.

The wire surfaces were evaluated before and after aging in the oral medium. For this purpose, 5 mm was sectioned from the most curved portion of each arch for evaluation by scanning electron microscopy (SS-550 Superscan, Shimadzu Biotech, Japan) at ×100 magnification.

Two blinded evaluators established the following scores for biofilm accumulation: 0 = total absence of debris; 1 = some debris, involving less than one-quarter of the image analyzed; 2 = moderate presence of debris, involving one-quarter of the image; 3 = the presence of a large quantity of debris, involving three-quarters of the image analyzed.The scores were compared by means of the kappa test; the discrepant results were discussed until a consensus was reached.^14,18^

**Figure 1:**
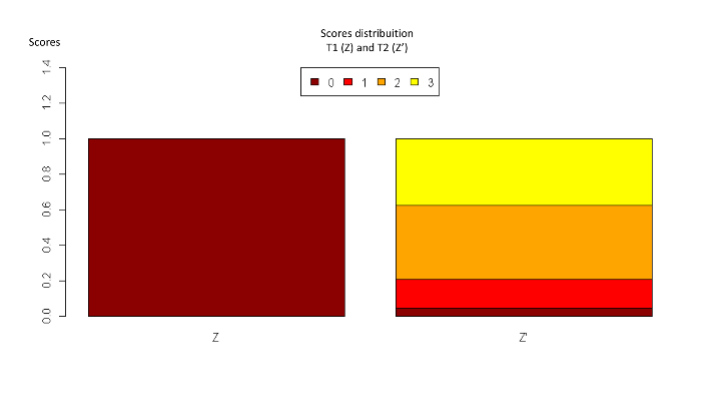
Distribution of general scores (all the brands) before and after exposure to the oral medium.

The three-point bending test before and after aging was adopted from the ISO 15.841 standard specification (Dentistry – Wires for use in orthodontics - INTERNATIONAL ORGANIZATION FOR STANDARDIZATION, 2006). The archwires were placed in a device containing four stainless steel self-ligating brackets (Morelli, São Paulo, Brazil), Roth prescription, 0.22” channel, for mandibular incisors, with 0° torque, and 0° angulation (Figure 1). The tests were performed in a universal testing machine (EMIC ® DL 1000, São José dos Pinhais, PR, Brazil) with archwire segments measuring 30 mm.

The three-point bending test was used, with a load cell of 50 N (5 kgf) and readout resolution of 0.01 N (1 gf). The constant temperature of 37±1°C was standardized by means of an oven (Biopar, Porto Alegre, RS, Brazil).

The distance between the two brackets was 10 mm (Figure 1), and the tests were conducted at a crosshead speed of 2.0 mm/min. The deflection forces were recorded at the following levels: 0.5, 1, 2, and 3 mm. The test began at a level of 3.1 mm of flexion.

Regarding the exposure of the archwires to the oral medium, six new archwires were used, corresponding to the first six arches, as received from the manufacturers. These were subjected to aging in the oral medium of patients undergoing orthodontic treatment. In total, 12 patients (6 women and 6 men), Brazilians, Caucasians, who had Class I and II malocclusions, complete dentition with exception of the third molars, each used two archwire samples. The volunteers’ age range was 12‒30 years. The patients were selected in accordance with the following inclusion criteria: moderate crowding, absence of anterior cross bite, severe deep bite, any mechanism adapted to orthodontic mechanics for correction of deep bite, no history of medical problems or use of medications, and no gingival problems.

Each patient randomly received two archwires of different brands: one in the maxillary, and one in the mandibular arch. Randomization was performed by a computer program (Microsoft Excel).

The archwires remained in the oral cavity of these patients for 4 weeks, when the archwires were re-evaluated with respect to deactivation force and biofilm accumulation.

### Statistical analysis

The kappa test was performed to evaluate the agreement between the two evaluators in the biofilm accumulation scores.

Distribution of the presence of biofilm among the 4 commercial brands, before and after exposure, was verified by means of the Wilcoxon test, and the Kruskal-Wallis test was used to evaluate the quantity of biofilm accumulation in each brand.

The mechanical test results were compared by means of three-way ANOVA – in terms of archwire brand, deflection and exposure to oral medium ‒ for repeated measures, and Bonferroni post-hoc test.

The correlation of biofilm accumulation and deactivation force was evaluated by the Spearman test by considering deflection at 2 mm.

The tests were analyzed at a significance level of 5%.

## Results

The kappa coefficient of agreement between the examiners with regard to biofilm accumulation was 0.74 (P<0.05).

The Wilcoxon and Kruskal-Wallis tests showed significant biofilm accumulation after exposure to the oral medium. Figure 1 illustrates the general distribution of the scores before and after aging (P<005).

**Figure 2:**
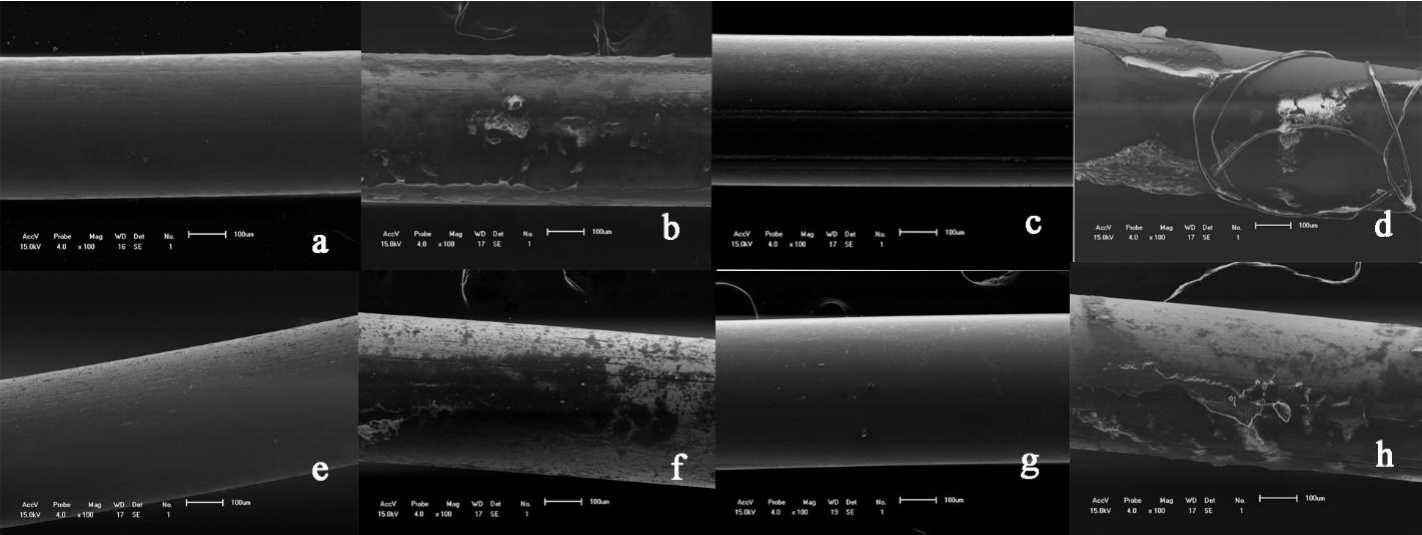
Biofilm accumulation before and after exposure to the oral medium at ×100 magnification.

The archwire surfaces presented moderate to severe biofilm deposition after exposure to the oral medium in comparison with T0 (Figure 2a-2h). All the brands presented this statistically significant behavior (Table 1).

**Table 1:** Descriptive analysis of the proportion of biofilm accumulation scores for the four different commercial brands

**Brands**	**Morelli**	**Orthometric**	**GAC**	**ORMCO**
**Scores**	**T1**	**T1**	**T1**	**T1**
**Zero**	100%	100%	100%	100%
**1**	0%	0%	0%	0%
**2**	0%	0%	0%	0%
**3**	0%	0%	0%	0%
**Scores**	**T2**	**T2**	**T2**	**T2**
**Zero**	0%	0%	0%	17%
**1**	33%	17%	0%	0%
**2**	0%	50%	67%	50%
**3**	67%	33%	33%	33%

0 = total absence of debris; 1 = some debris that involved less than one-quarter of the image analyzed; 2 = moderate presence of debris involving one-quarter of the image; 3 = presence of a large quantity of debris that involved three-quarters of the image analyzed.

**Figure 3:**
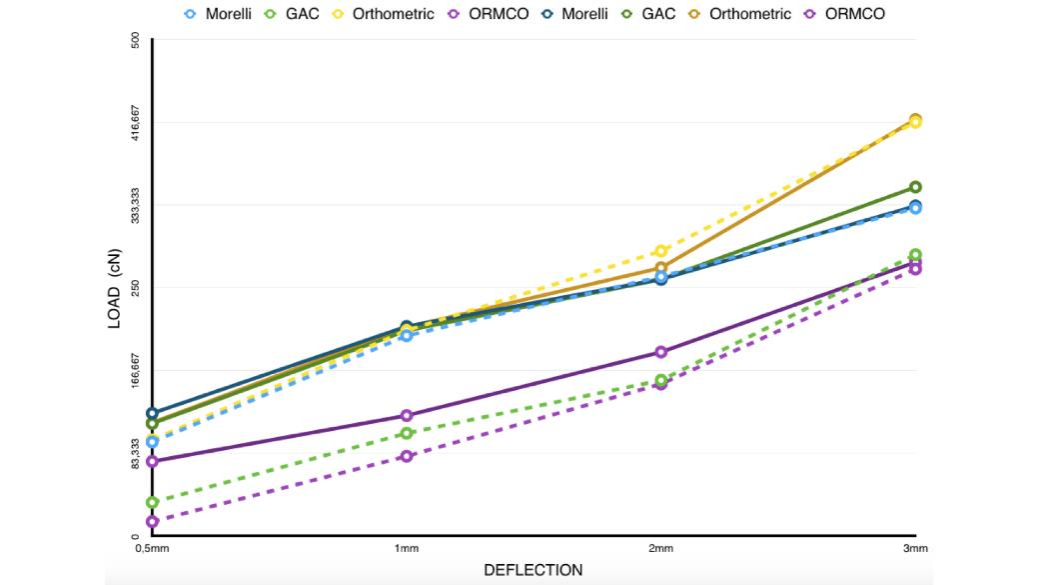
Representation of force generated in the deactivation of the NiTi archwires before and after exposure.

Three-way ANOVA showed statistically significant difference in the levels of force after exposure to the oral medium (P<0.05) and between the brands studied (Figure 3, Table 2).

**Table 2:** Mean and standard deviation of samples of four brands for the 3-point bending test on the same line, different lowercase letters represent statistical significance (P<0.05)

**T0**	**MORELLI**	**GAC**	**ORTHOMETRIC**	**ORMCO**
**Force / 0.5 mm (cN)**	123.7 (±15)^a,A^	113.4 (±34)^a,A^	114.2 (±25)^a,A^	75.16 (±15)^a,A^
**Force / 1 mm (cN)**	211.2 (±10)^a,A^	207 (±33)^b,A^	210.5 (±37)^a,A^	121.4 (±22)b,A
**Force / 2 mm (cN)**	258.8 (±11)^a,A^	258.6 (±31)^b,A^	270.3 (±35)^a,A^	185.4 (±21)^b,A^
**Force / 3 mm (cN)**	332.5 (±24)^a,A^	351.6 (±36)^b,A^	419.6 (±28)^c,A^	275.9 (±14)^c,A^
**T1**				
**Force / 0.5 mm (cN)**	94.74 (±65)^a,A^	33.89 (±58)^ab,B^	97 (±39)^a,A^	14.5 (±37)^b,B^
**Force / 1 mm (cN)**	201.8 (±49)^a,A^	103.6 (±58)^a,B^	207.2 (±49)^a,A^	80.44 (±21)^b,B^
**Force / 2 mm (cN)**	261.4 (±27)^a,A^	157 (±49)^a,B^	287.1 (±34)^a,A^	153.2 (±17)^b,B^
**Force / 3 mm (cN)**	330.3 (±39)^a,A^	283.5 (±12)^a,B^	416.9 (±28)^b,A^	269 (±14)^c,A^

In the same column, different capital letters represent statistical difference before and after exposure to the oral medium (P<0.05). 1cN ~1g / n=6

There was an increase in force with an increase in deflection for all the archwires, without statistical significance only for the Morelli brand.

The Spearman test indicated a low correlation between biofilm accumulation and deflection force for the Morelli (R2=0.132 and P=0.683) and Orthometric (R2=0.308 and P=0.330) brands. On the other hand, the GAC (R=0.767 and P=0.004) and ORMCO (R=0.725 and P=0.008) brands presented statistically significant correlation between these variables, demonstrating that biofilm accumulation influenced the manifestation of deactivation force.

## Discussion

The evaluation of biofilm accumulation and deactivation force of NiTi archwires before and after exposure to the oral medium simulated a condition that has hardly been explored in the mechanical studies of orthodontic archwires. Generally, the properties of archwires are evaluated in a laboratory, using models without considering the presence of biofilm.^3,16,20,21^

In the present study, it became clear that the accumulation of biofilm interferes with the dissipation of the archwire force and might consequently reduce the effectiveness of movement during orthodontic treatment.

Studies on the variables that affect the mechanical properties of orthodontic archwires and accessories after exposure to the oral cavity have demonstrated that the superficial aspect of the archwires influenced the original performance of the wire.^19^

The acidic pH produced by the bacteria present in the debris might increase their roughness, hardness and friction between the wire and bracket channel,^6,17,21^ thereby contributing to a reduction in the deactivation force of the archwires. In the present study, there was greater biofilm accumulation on the Morelli brand, which had ¾ of its samples covered with debris, while the other three brands, Ormco, GAC, and Orthometric, presented accumulation in ½ of the samples. These data corroborate those in the literature, demonstrating a significant increase in the degree of debris and in surface roughness of orthodontic archwires after exposure to the oral medium.^6,15,17,18^

The Morelli and Orthometric brand archwires showed no significant differences in the deactivation force after exposure, whereas the GAC and Ormco brands presented a significant reduction in the activation force. However, the force threshold continued to be within values required for a favorable orthodontic response for the GAC and Ormco brands and continued a little high for the MORELLI and Orthometric brands.

There was a significant correlation between the presence of biofilm and a reduction in force in the ORMCO and GAC archwires. This did not occur for the Morelli and Orthometric archwires, which might be related to the fact that the force thresholds of these archwires were higher, and thus, the frictional force generated by the presence of biofilm was no sufficient to reduce the deactivation force in these brands.

Jaber et al^22^ found that although cariogenic and erosive substances had increased the surface roughness of CuNiTi archwires, this roughness did not significantly increase the friction between the wire and self-ligated bracket. This might have occurred due to the difference in the methodology, as the samples were exposed to artificial saliva in vitro that is different from the present study, in which the archwires were exposed in vivo.

The Ormco archwire used in this study was the only heat-activated type. Therefore, as expected, it presented force in lower levels of dimension than that of the superelastic NiTi analogs.^2,23^ Most probably the heat-activated superelastic archwires of the other brands, equivalent in dimension, could present lower levels of forces, as we found in a previous study.^24^

Although we used a three-point bending test, we sought to adapt it to a clinical situation by using a model with self-ligating brackets coupled to a universal testing machine, so that the factors such as the type of bracket^25^ and the mechanism of ligation influenced the deactivation of the NiTi archwire.^26^

Nucera et al^25^ observed that the conventional three-point bending test resulted in the deactivation forces 40‒70% lower in comparison with those of the three-point tests performed with conventional and self-ligating brackets adapted to the traction device, demonstrating that the adaptation of brackets to the test resulted in data closer to those of the clinical reality. This is because the classical three-point test does not take into account the frictional force of the brackets, thereby reducing the interference of this variable. Nevertheless, the clinical extrapolation of these laboratory models, such as that of the present study, must adopt a ranking system for the comparison of the results.^9^

The different behaviors of the archwires are related to the complexity of the manufacturing process, including the cold work, heat treatment, and composition of the archwires, together with variability among the lots tested.^27-29^

The period of exposure to the oral medium in this study was one month, which indicated great influence on the behavior of the archwires. Eventually, a leveling archwire remains in the patient’s mouth for 3 months, with greater exposure and corrosion. Therefore, orthodontists must be aware of this factor when making a decision to keep the same orthodontic archwire in the patient’s mouth for a long period. In addition to the impact on the levels of force, more detailed studies are recommended on corrosion and the release of ions.

In the present study, the influence of biofilm collection within the brackets was not evaluated. For the same reason as pointed out here for the reduction of load by virtue of friction “robbing” part of the archwire force, even greater interference could be expected in studies that also submit the brackets to “aging” in the oral environment.

## Conclusion

The present study demonstrated that the archwire surfaces had moderate to severe biofilm deposition after exposure to the oral medium.

The deactivation force test showed statistically significant differences in the levels of force after exposure to the oral medium (P<005) and between the brands studied.

Exposure to the oral medium for one month might cause significant changes in the dissipation of forces of orthodontic NiTi archwires, resulting from biofilm accumulation.

## Acknowledgements

None.

## Ethics Approval

The present study was approved by the Research Ethics Committee of the State University of Maringá (UEM) (CAAE: 43121915.2.0000.0104), and the patients signed a term of free and informed consent.

## Competing Interests

The authors declare no conflict(s) of interest related to the publication of this work.

## Funding

This study was conducted with the resources of the authors.

## Authors’ Contributions

DMS wrote and conducted the study; ALR analyzed and interpreted the data; DN contributed to the discussion section; SS collected the database; and RCP guided the article and the authors.
